# Attosecond pulse retrieval from noisy streaking traces with conditional variational generative network

**DOI:** 10.1038/s41598-020-62291-6

**Published:** 2020-04-01

**Authors:** Zheyuan Zhu, Jonathon White, Zenghu Chang, Shuo Pang

**Affiliations:** 1 CREOL, The College of Optics and Photonics, University of Central Florida, Orlando, FL 32816 United States; 2 Department of Physics, University of Central Florida, Orlando, FL 32816 USA

**Keywords:** Ultrafast lasers, Ultrafast photonics

## Abstract

Accurate characterization of an attosecond pulse from streaking trace is an indispensable step in studying the ultrafast electron dynamics on the attosecond scale. Conventional attosecond pulse retrieval methods face two major challenges: the ability to incorporate a complete physics model of the streaking process, and the ability to model the uncertainty of pulse reconstruction in the presence of noise. Here we propose a pulse retrieval method based on conditional variational generative network (CVGN) that can address both demands. Instead of learning the inverse mapping from a streaking trace to a pulse profile, the CVGN models the distribution of the pulse profile conditioned on a given streaking trace measurement, and is thus capable of assessing the uncertainty of the retrieved pulses. This capability is highly desirable for low-photon level measurement, which is typical in attosecond streaking experiments in the water window X-ray range. In addition, the proposed scheme incorporates a refined physics model that considers the Coulomb-laser coupling and photoelectron angular distribution in streaking trace generation. CVGN pulse retrievals under various simulated noise levels and experimental measurement have been demonstrated. The results showed high pulse reconstruction consistency for streaking traces when peak signal-to-noise ratio (SNR) exceeds 6, which could serve as a reference for future learning-based attosecond pulse retrieval.

## Introduction

The generation of isolated attosecond extreme ultraviolet (XUV)/soft X-ray pulses is a milestone toward investigating the ultrafast electron dynamics on its natural time scale. Accurate temporal and spectral characterization of these XUV pulses is a crucial step in attosecond pump-probe experiments^1^. Because the spectrum of the XUV pulses can be measured relatively easily with a spectrometer, knowing its spectral phase will enable a complete reconstruction of the XUV pulse in both time- and frequency-domain. Adapted from an iterative femtosecond pulse retrieval method FROG (Frequency Resolved Optical Gating), Frequency-resolved optical gating for complete reconstruction of attosecond bursts (FROG-CRAB) has been widely used for phase retrieval from attosecond streaking traces^2^. Compared to its femtosecond counterpart FROG, attosecond phase retrieval suffers from both experimental and theoretical challenges. The photon flux of attosecond soft X-ray is much lower than that in a typical FROG experiment, giving rise to higher statistic noise in the streaking trace. Currently the effect of noise on the error and uncertainty of retrieved pulse is yet to be understood, and there lacks a guideline on the signal-to-noise ratio (SNR) requirement for accurate pulse retrieval. In addition, phase retrieval of ultra-broadband XUV/X-ray pulses requires a more thorough theoretic description of the photoelectron wave packet during the streaking process, including its energy and angular distribution, as well as its interaction with the laser field, which are either simplified or omitted in existing pulse retrieval methods^3^.

Accurate physics descriptions of the attosecond streaking process and phase retrieval algorithms have been developed synergistically to enable the characterization of broadband pulses with durations shorter than the atomic unit of time. PROOF^4^, Volkov transform generalized projection algorithm (VTGPA)^5,8^ and genetic algorithms^4,9,10^ consider a phase gate that depends on energy and time, obviating the Central Momentum Approximation in FROG-CRAB. Incorporating the angular distribution of photoelectrons^5,6^ have led to a more accurate transition dipole moment that matches the streaking process in experiments. These refined streaking models demand optimization-based retrieval methods beyond the scope of principle component generalized projections^7^ used in FROG-CRAB. Recently, deep neural networks (DNNs)^11,12^ have also been applied to learn the inverse mapping of these sophisticated streaking models. However, these methods are insufficient to address the aforementioned challenges, mainly because their output is a deterministic solution given an input streaking trace, and therefore unable to capture the intrinsic pulse variance due to the noise. Moreover, the interaction between photoelectron and the laser field is not fully included in existing models^3^. In this work, we propose a conditional variation generative network (CVGN) to model the distribution of all possible pulses that satisfy a streaking trace corrupted by Poisson noise. We have incorporated a refined streaking model that considers the photoelectron angular distribution^5^ and additional phase delay from Coulomb-laser coupling^3^. The results quantitatively demonstrate the uncertainty in the pulse domain, which arises naturally from the experimental noise, and provide an guideline on the required SNR for future streaking experiments.

## Theory

### Attosecond streaking trace generation

The streaking trace in our experiment is a series of photoelectron spectra $y(|\overrightarrow{k}|,\tau )$ generated from the interaction between an attosecond XUV/X-ray pulse ${E}_{X}(t)$ and a femtosecond infrared (IR) pulse ${E}_{IR}(t)$ in Helium with various time delays $\tau$. In an attosecond streaking experiment, both XUV/X-ray and IR pulses are polarized along the direction of the time-of-flight (TOF) spectrometer^5^, so the interaction can be calculated as scalar product. Once the gas atoms with photoionization potential ${I}_{p}$ absorb the energy from an XUV/X-ray pulse, electrons transit from ground state to the excited state (with momentum $\overrightarrow{k}$) with a transition amplitude $a$ described by Eq. (1) in atomic units^5^. Here we apply the Strong-Field Approximation (SFA)^13^ that is valid for photoelectron energy larger than 40 eV^6^.

1 $$a(\overrightarrow{k},\tau )=a(|\overrightarrow{k}|,\theta ,\tau )=-\,{\mathbb{i}}\int {\Psi }_{e}(t-\tau ,\theta )\exp ({\mathbb{i}}{\phi }_{G}(\overrightarrow{k},t))\exp ({\mathbb{i}}({|\overrightarrow{k}|}^{2}/2+{I}_{p})t)dt,$$

where ${\phi }_{G}(\overrightarrow{k},t)$ is a phase gating created by the IR dressing field,

2 $${\phi }_{G}(\overrightarrow{k},t)={\phi }_{G}(|\overrightarrow{k}|,\theta ,t)=-\,{\int }_{t}^{\infty }(|\overrightarrow{k}|A(t{\prime} )\cos \,\theta +{|A(t{\prime} )|}^{2}/2)dt{\prime} .$$

$A(t)=-\,{\int }_{-\infty }^{t}{E}_{IR}(t{\prime} )dt{\prime}$ is the vector potential of the IR field, which is also along the direction of TOF spectrometer; $\theta$ is the angle between the photoelectron momentum, $\overrightarrow{k}$, and the IR field polarization direction. Notice that if $\overrightarrow{k}$ is approximated by a single value scalar $|{\overrightarrow{k}}_{0}|$ corresponding to the central momentum, Eq. (2) reduces to the conventional FROG-CRAB method^2^, which only applies to a phase gating independent on $\overrightarrow{k}$ and hence reduces the accuracy when retrieving the phase of ultra-broadband XUV/X-ray spectrum^4^.

In atomic unit, the wave packet of the induced photoelectron, ${\Psi }_{e}$, is represented by

3 $${\Psi }_{e}(t,\theta )\propto {\int }_{-{\rm{\infty }}}^{+{\rm{\infty }}}|{\mathop{E}\limits^{ \sim }}_{X}(\epsilon )|{e}^{{\mathbb{i}}{\varphi }_{X}(\epsilon )}d(\epsilon ,\theta ){e}^{-{\mathbb{i}}\epsilon t}d\epsilon .$$

here $|{\tilde{E}}_{X}({\epsilon })|$ and ${\varphi }_{X}$ are amplitude and phase of the XUV/X-ray electric field. ${\epsilon }$ is the photon energy that equals the angular frequency in atomic units. Since the power spectrum ${|{\tilde{E}}_{X}({\epsilon })|}^{2}$ can be directly measured, the main task of the XUV/X-ray pulse characterization is to determine the spectral phase ${\varphi }_{X}$. Some FROG-based phase retrieval methods^2,4^ assume a constant transition dipole matrix element $d$ to simplify the streaking model. For broadband pulses, the energy dependency of the magnitude and phase of $d$ must be included. In addition, photoelectron distributions in streaking experiments are angular dependent^14^, and the electron wave packet is subject to additional phase shifts from Coulomb-laser coupling^3,15^. This gives rise to an energy- and angular-dependent dipole matrix element, $d({\epsilon },\theta )$, which is expressed as

4 $$d({\epsilon },\theta )\propto \sqrt{f(\theta )\sigma ({\epsilon })}\exp ({\mathbb{i}}\eta ({\epsilon }))\exp ({\mathbb{i}}\delta ({\epsilon })),$$

where ${\epsilon }$ denotes the energy; $\sigma ({\epsilon })$ is the photoionization cross-section of Helium obtained from^16^; $\eta ({\epsilon })={\rm{\arg }}\{\Gamma (2-{\mathbb{i}}/\sqrt{2({\epsilon }-{I}_{p})})\}$ is the phase delay from the Coulomb potential^3^, $\Gamma [\,\cdot \,]$ representing the complex gamma function; $\delta ({\epsilon })={\int }_{0}^{{\epsilon }-{I}_{p}}{(2{{\epsilon }}^{\text{'}})}^{3/2}(2-\,\mathrm{ln}({{\epsilon }}^{\text{'}}{T}_{IR}))d{\epsilon }\text{'}$ is the phase delay from IR dressing laser^15^, where ${T}_{IR}=2\pi c/{\lambda }_{c}$ is the oscillating period of the IR field in atomic unit. The angular dependency of the photoelectron $f(\theta )=1+\beta (3{\cos }^{2}\theta -1)/2$, where $\beta$ is a parameter that accounts for the asymmetry distribution of the photoelectrons ionized from different gas atoms, and $\beta$ = 1 for Helium gas^17^. The measured spectrogram is the integral of intensity over all the directions of $\overrightarrow{k}$, which falls within an angular range of [0, ${\theta }_{max}$) against the TOF detector.

5 $$y(|\overrightarrow{k}|,\tau )={\int }_{\theta =0}^{{\theta }_{max}}{|a(\overrightarrow{k},\tau )|}^{2}\,\sin (\theta )d\theta .$$

In the forward model of the streaking process, *y* is discretized as a two-dimensional array ${\bf{y}}$.

### Trace retrieval with conditional variational generative network

The streaking traces ${\bf{y}}$ measured from experiments contain statistic noise due to the limited photon flux and data acquisition time, which introduces uncertainty to the mapping from the pulse domain, ${\bf{x}}$, to the streaking trace domain, ${\bf{Y}}$. Conditional variational generative network (CVGN)^18^ models the distribution of possible XUV/X-ray and IR pulse parameters, ${\bf{x}}$, given a streaking trace, ${\bf{y}}$, via the parameterization of the posterior distribution by the latent variable, ${\bf{z}}$

6 $${p}_{\gamma }({\bf{x}}|{\bf{y}})=\int {p}_{\gamma }({\bf{x}}|{\bf{z}},{\bf{y}}){p}_{\gamma }({\bf{z}}|{\bf{y}})d{\bf{z}},$$

where ${p}_{\gamma }({\bf{z}}|{\bf{y}})$ is the conditional prior distribution of ${\bf{z}}$, given an input streak ${\bf{y}}$. The retrieved pulse parameters, ${\bf{x}}$ is sampled from the distribution ${p}_{\gamma }({\bf{x}}|{\bf{z}},{\bf{y}})$. Both ${p}_{\gamma }({\bf{z}}|{\bf{y}})$ and ${p}_{\gamma }({\bf{x}}|{\bf{z}},{\bf{y}})$ are assumed to be multivariate Gaussians with diagonal covariance matrix. ${p}_{\gamma }({\bf{z}}|{\bf{y}})={\mathscr{N}}({\bf{z}};{{\boldsymbol{\mu }}}_{{\bf{z}}}^{(\gamma )}({\bf{y}}),diag({[{{\boldsymbol{\sigma }}}_{{\boldsymbol{z}}}^{(\gamma )}({\bf{y}})]}^{2}))$, ${p}_{\gamma }({\bf{x}}|{\bf{z}},{\bf{y}})={\mathscr{N}}({\bf{x}};{{\boldsymbol{\mu }}}_{{\bf{x}}}^{(\gamma )}({\bf{y}},{\bf{z}}),\beta {\bf{I}})$, where the mean and variance parameters ${{\boldsymbol{\mu }}}_{{\bf{z}}}^{(\gamma )}({\bf{y}})$, ${{\boldsymbol{\mu }}}_{{\bf{x}}}^{(\gamma )}({\bf{y}},{\bf{z}})$ and ${{\boldsymbol{\sigma }}}_{{\bf{z}}}^{(\gamma )}({\bf{y}})$ are implemented by neural networks with parameter $\gamma$^19^, and $\beta$ is a hyper parameter that determines the covariance of the posterior distribution.

The training process of CVGN maximizes the joint log-likelihood $\log \,{p}_{\gamma }({\bf{x}}|{\bf{y}})=\mathop{\sum }\limits_{i=1}^{N}\,\log \,{p}_{\gamma }({{\bf{x}}}_{i}|{{\bf{y}}}_{i})$ of observing the pulse parameters vs trace pairs $\{({{\bf{x}}}_{i},{{\bf{y}}}_{i}),i=1,\ldots ,N\}$ in the dataset. Due to the intractable integral in Eq. (5), a lower bound of the log-likelihood is used as the objective function, $\, {\mathcal L}$, with the introduction of a recognition distribution $\,{q}_{\kappa }({\bf{z}}|{\bf{x}},{\bf{y}})$^20^

7 $$\log \,{p}_{\gamma }({{\bf{x}}}_{i}|{{\bf{y}}}_{i})\ge -\,KL({q}_{\kappa }({\bf{z}}|{{\bf{x}}}_{i},{{\bf{y}}}_{i})||{p}_{\gamma }({\bf{z}}|{{\bf{y}}}_{i}))+{E}_{{q}_{\kappa }({\bf{z}}|{{\bf{x}}}_{i},{{\bf{y}}}_{i})}(\log \,{p}_{\gamma }({{\bf{x}}}_{i}|{\bf{z}},{{\bf{y}}}_{i}))\,:\,= {\mathcal L} ,$$

where ${q}_{\kappa }({\bf{z}}|{{\bf{x}}}_{i},{{\bf{y}}}_{i})$ captures the latent distribution conditioned on both the streaking trace and pulse parameters. If we model ${q}_{\kappa }({\bf{z}}|{{\bf{x}}}_{i},{{\bf{y}}}_{i})$ as a multivariate Gaussian with diagonal covariance matrix ${\mathscr{N}}({\bf{z}};{{\boldsymbol{\mu }}}_{{\bf{z}}}^{(\kappa )}({\bf{x}},{\bf{y}}),diag({[{{\boldsymbol{\sigma }}}_{{\boldsymbol{z}}}^{(\kappa )}({\bf{x}},{\bf{y}})]}^{2}))$, whose mean and variance are also implemented by neural networks with parameter $\kappa$, the objective function to maximize has a closed form

8 $$ {\mathcal L} =-\,\mathop{\sum }\limits_{j=1}^{M}\left(\log \,\frac{{\sigma }_{ij}^{(\kappa )}}{{\sigma }_{ij}^{(\gamma )}}+\frac{{({\mu }_{ij}^{(\gamma )}-{\mu }_{ij}^{(\kappa )})}^{2}+{\sigma }_{ij}^{(\gamma )2}}{2{\sigma }_{ij}^{(\gamma )2}}-\frac{1}{2}\right)-\frac{1}{\beta L}\mathop{\sum }\limits_{l=1}^{L}{({{\bf{x}}}_{i}-{{\boldsymbol{\mu }}}_{{\bf{x}}}^{(\gamma )}({{\bf{z}}}_{l},{{\bf{y}}}_{i}))}^{2},$$

where ${\sigma }_{ij}^{(\kappa )}$ denotes the $j$-th index of the *M*-element vectors ${{\boldsymbol{\sigma }}}_{{\bf{z}}}^{(\kappa )}({{\bf{x}}}_{i},{{\bf{y}}}_{i})$; similar notations are applied to ${\sigma }_{ij}^{(\gamma )}$, ${\mu }_{ij}^{(\kappa )}$ and ${\mu }_{ij}^{(\gamma )}$. The expectation in Eq. (6) is approximated by sampling $L$ instances of ${\bf{z}}$ from the distribution ${q}_{\kappa }({\bf{z}}|{{\bf{x}}}_{i},{{\bf{y}}}_{i})$ as $\{{{\bf{z}}}_{l}:l=1,\ldots ,L\}$.

### CVGN construction and model training

The XUV/X-ray and IR pulses were represented by a set of 9 parameters, denoted as the vector ${\bf{x}}$. The first 5 elements describe the XUV/X-ray spectral phase. Because the spectrum of XUV pulse can be measured relatively easily from the experiment, the XUV/X-ray field can be uniquely determined by its spectral phase ${\varphi }_{X}({\epsilon })$, which was expressed as a 5^th^ order Taylor series in atomic unit,

9 $${\tilde{E}}_{X}({\epsilon })=\sqrt{{S}_{X}({\epsilon })}\exp {\mathbb{i}}\mathop{\sum }\limits_{i=1}^{5}{k}_{i}{{\epsilon }}^{i}.$$

here ${S}_{X}({\epsilon })={S}_{e}({\epsilon }-{I}_{p})/\sigma ({\epsilon })$ is the spectral density of the XUV/X-ray pulse, which can be obtained from the experimentally measured photoelectron spectrum ${S}_{e}({\epsilon }-{I}_{p})$ by correcting for the Helium cross-section $\sigma ({\epsilon })$. The photoelectron wave packet ${\Psi }_{e}(t)$ was then obtained by adding the energy- and angular-dependent dipole moment $d({\epsilon },\theta )$ (Eq. (3)) to the complex XUV/X-ray spectrum ${\tilde{E}}_{X}({\epsilon })$, and Fourier transformed into time-domain for streak calculation (Eqs. (1–4)). The time-domain XUV/X-ray pulse was constructed directly from the Fourier transform of Eq. (8). In addition, 4 elements representing the carrier envelop phase ${\phi }_{CEP}$, central wavelength ${\lambda }_{0}$, pulse duration ${\tau }_{IR}$, and peak field strength ${A}_{IR}$ were used to characterize the IR dressing pulse in the time domain^11^, expressed in Eq. (9)

10 $${\tilde{E}}_{IR}(t)={A}_{IR}\exp \left(-2\,\mathrm{ln}(2){\left(\frac{t}{{\tau }_{IR}}\right)}^{2}\right)\exp ({\mathbb{i}}(\frac{2\pi c}{{\lambda }_{0}}t+{\phi }_{CEP})).$$

The training data were generated by adding noise to the ideal, noise-free traces from the physics model. We first created 10000 ideal traces with random pulse parameters, ${\bf{x}}$, and normalized their intensities to the range between 0 and 1. Poisson noise was added to each ideal trace ${{\bf{y}}}_{0}$ to simulate noisy traces in experiments

11 $${\bf{y}}\sim Poisson(\lambda {{\bf{y}}}_{0}),$$

where the parameter $\lambda$ is the average peak count of the Poisson distribution. The training data contained a mixture of 5 different Poisson noise levels, $\lambda$ = 10, 32.5, 55, 77.5 and 100. An additional 1000 ideal traces were used to test the trained model, which consisted of 10 different Poisson noise levels, $\lambda$, ranging from 5 to 100.

The structure of CVGN used for attosecond streaking trace retrieval was constructed according to that in ref. ^19^. It is worth noting that the output (label) of this CVGN ${\bf{x}}$ is a vector with the 9 pulse parameters, instead of the real and imaginary part of the XUV spectrum in ref. ^19^. After the training, 25 instances of pulse parameters $\hat{{\bf{x}}}$ were retrieved from the distribution ${p}_{\gamma }({\bf{x}}|{\bf{z}},{\bf{y}})$ by sampling $\{{{\bf{z}}}_{l}:l=1,\ldots ,25\}$ from the conditional prior distribution ${p}_{\gamma }({\bf{z}}|{\bf{y}})$. The time- and frequency-domain XUV pulses were then reconstructed from these pulse parameters.

## Results and discussions

**Figure 1:**
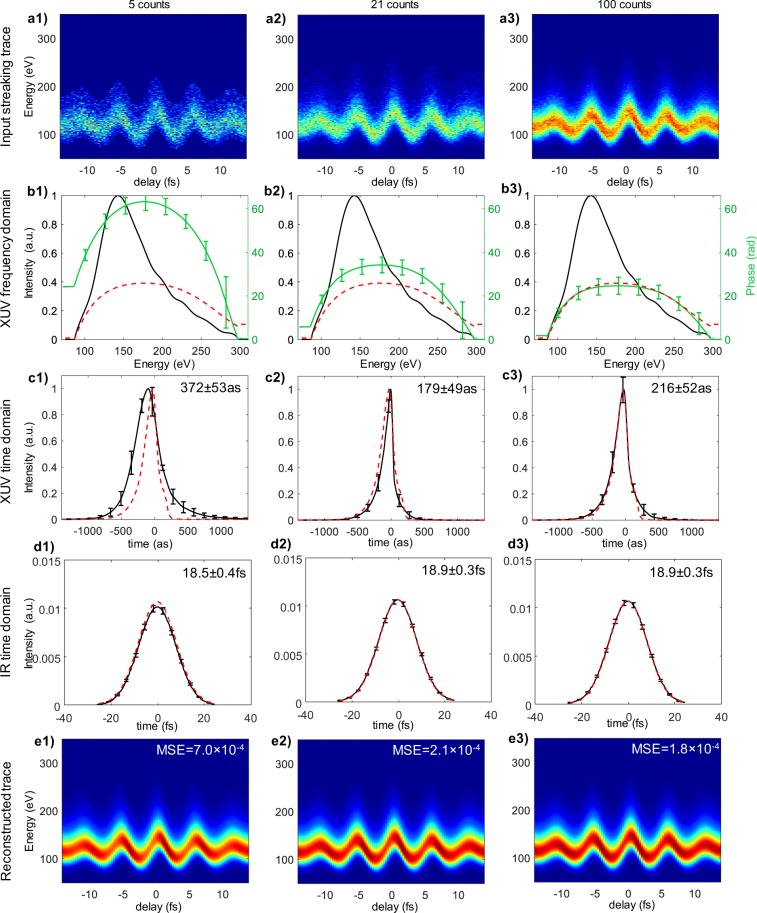
Pulse retrieval from a simulated streaking trace. (**a**) Input streaking traces. (**b**,**c**) Retrieved XUV spectral phase (**b**) and temporal envelop (**c**). **(d**) Reconstructed IR pulse in time domain. The dashed red curves on (**b–d**) indicate the ground truth. Error bars on (**b–d**) represent the variance of the retrieved pulse instances. Numbers on the upper right corner indicate FWHM of retrieved pulse. (**e**) Reconstructed streaking trace from the retrieved pulses. The MSE indicates the error between the reconstructed streaking trace and the ground truth.

In this section, the retrieved XUV/X-ray pulses from both test dataset and experimental streaking traces using a trained CVGN are presented to show the accuracy at various noise levels. Figure 1(a1–a3) shows three simulated streaking traces from the same noise-free test trace with $\lambda$ = 5, 21 and 100. For each trace, 25 instances of the pulse parameter set, $\hat{{\bf{x}}}$, were retrieved from the posterior distribution ${p}_{\gamma }({\bf{x}}|{\bf{y}},{\bf{z}})$. The frequency (Fig. 1(b1–b3)) and time-domain (Fig. 1(c1–c3)) XUV pulses, along with the time-domain envelop of IR pulses (Fig. 1(d1–d3)), were reconstructed from these retrieved pulse parameters. Streaking traces (Fig. 1(e1–e3)) were then generated from the retrieved XUV and IR pulses using the physical model in Eqs. (1–4). Error bars on Fig. 1(b–d) represent the variance of the 25 instances of retrieved XUV pulse.

**Figure 2:**
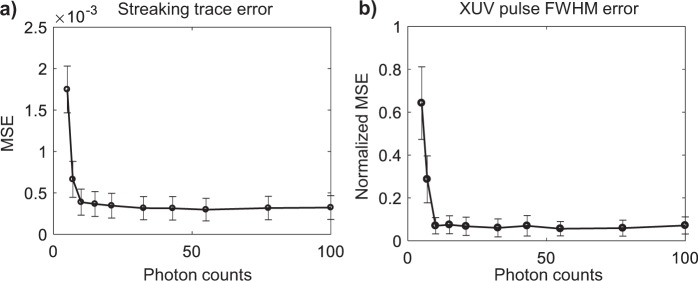
MSE of (**a**) reconstructed streaking trace (**b**) FWHM of retrieved time-domain XUV pulse at various Poisson noise levels. The error bars represent the MSE fluctuation within the whole test dataset.

The reconstructed streaking traces and the full-width-at-half-maximum (FWHM) of the time-domain XUV pulse were compared with the ground truth. The mean squared error (MSE) of the 25 reconstruction instances was used as the error metric to evaluate the performance under various noise levels. The reconstructed and ideal streaking traces were both normalized to facilitate the comparison among various $\lambda$. For the errors on pulse duration, the MSE was normalized by the FWHM of the XUV ground truth, which was 209as in the simulation. Figure 2(a,b) plot the MSE on the reconstructed streaking traces and pulse FWHM with respect to $\lambda$, averaged over the whole test dataset.

The results show that as the average peak count, $\lambda$, exceeds 32.5, the MSE of the reconstructed streaking trace decreased and remained below 3.5 × 10^−4^. The MSE of pulse duration also decreased from ~80% ($\lambda$ = 5) down to 6% ($\lambda =32.5$). The results suggest an average peak Poisson SNR of at least 6 to achieve satisfactory pulse retrieval. It is worth noting that for low photon count (5 counts), the retrieved XUV field exhibits increased bias in pulse profile, which is an indication of strong regularization^21^. This is the effect of using the training data with mixed noise level, which can be reduced by training with traces with same noise level or implementing additional mechanism to adjust the posterior distribution based on noise level. The retrieved time-domain IR envelop, on the other hand, is less affected by the noise. This is because IR envelop is represented by a Gaussian function with relatively few parameters, and is oversampled by 20 delays per cycle in the streaking measurements. Because of CVGN’s flexibility in capturing the distribution of solutions satisfying a physical model, it is not necessary to restrict the IR envelop to a Gaussian shape. We envision a complex IR envelop (amplitude and phase chirp) can be retrieved by using more parameters in describing the IR field.

**Figure 3:**
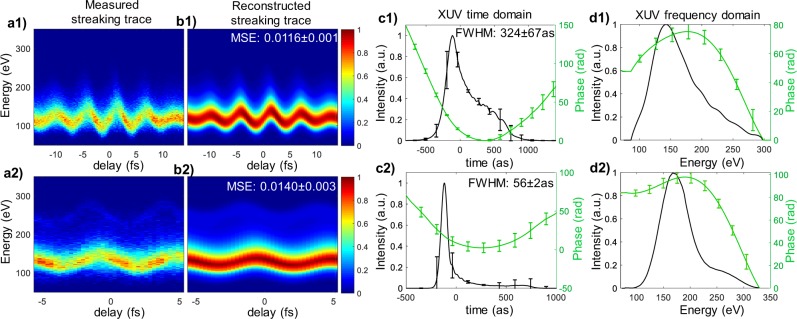
Retrieval of 2 experimental streaking traces with variational generative network. (**a**) Measured streaking trace. **(b**) Average of reconstructed streaking trace from 10 retrieved pulses. (**c**,**d**) Time and frequency domain of the retrieved XUV pulse. The error bars mark the standard deviation of the 10 instances.

To characterize the reconstruction performance under a realistic photon flux, we tested the trained variational generative network with experimentally measured streaking traces (Fig. 3(a)) in previous studies^22,23^, and retrieved 10 instances of pulse parameters from the posterior distribution ${p}_{\gamma }({\bf{x}}|{\bf{y}},{\bf{z}})$ given each trace ${\bf{y}}$. The time and frequency domain of the XUV pulse were reconstructed from these pulse parameters, and streaking traces were simulated for each retrieved pulse. Figure 3(b) shows the average of 10 reconstructed streaking traces generated from retrieved XUV pulses. Compared with the experimental trace (both traces were normalized to 0~1), the MSE was 0.0116 ± 0.0001 for trace 1, and 0.0140 ± 0.0003 for trace 2. The mean and standard deviation of retrieved pulse in the time and frequency domain are shown in Fig. 3(c,d). The average FWHM of the 10 retrieved pulses was 324 ± 67as for trace 1, and 56 ± 2 as for trace 2. The reconstructed pulses agree with previously reported PROOF results^22,23^ within the error margin.

## Conclusion

We have demonstrated the application of CVGN for phase retrieval from noisy streaking traces. The retrieved XUV pulse from a trained CVGN agrees with previously reported experimental results obtained from iterative PROOF method. The advantage of CVGN over deterministic ultrafast pulse retrieval DNNs lies in the flexibility to capture the pulse retrieval errors and uncertainties under various noise levels. Simulations using a refined streaking generation process and CVGN retrieval have demonstrated a Poisson peak SNR of over 6 can generate accurate pulse retrieval, which is indicative for SNR requirement in attosecond streaking experiments.
